# Multiple Walker breakdowns in magnetic multilayers

**DOI:** 10.1038/s41598-022-06275-8

**Published:** 2022-02-10

**Authors:** Joon Moon, Jaesung Yoon, Kitae Kim, Seong-Hyub Lee, Dae-Yun Kim, Sug-Bong Choe

**Affiliations:** 1 Department of Physics and Astronomy, Seoul National University, Seoul, 08826 Republic of Korea; 2 Department of Electrical and Computer Engineering, National University of Singapore, Singapore, 117582 Singapore

**Keywords:** Spintronics, Electronic and spintronic devices, Surfaces, interfaces and thin films

## Abstract

Herein, we report an exotic domain-wall dynamics showing double Walker breakdowns in magnetic multilayer films composed of two magnetic layers. Such multiple Walker breakdowns are attributed to the internal magnetic dipole field, which is antisymmetric on the domain walls of the lower and upper magnetic layers. A micromagnetic simulation shows four phases of the domain-wall dynamics, which result in a phase diagram with the phase boundaries of the double Walker breakdown fields. Such double Walker breakdowns lead to two minima in the variation of the domain-wall velocity, as often observed experimentally.

## Introduction

Recently, magnetic thin films have drawn great technological attention because of their prospects for use in next-generation memory and logic devices^1,2^. The data bits in these devices are stored in their magnetization states and/or ordering structures such as magnetic domains and domain walls (DWs). The device operation is therefore performed by switching between the magnetization states as done for the magnetic random-access memory and/or the displacement of magnetic structures as done for the magnetic racetrack memory^3,4^. These magnetization dynamics and their characteristics are determined by magnetic parameters such as perpendicular magnetic anisotropy, Dzyaloshinskii–Moriya interaction, and the spin–orbit coupling effect^5–9^. As these phenomena are generated at interfaces adjacent to magnetic layers, much effort has been devoted to controlling the interface properties in magnetic thin films and multilayered structures^10^.

For multilayered structures with multiple magnetic layers, the magnetic properties of each magnetic layer are largely determined by their own interfaces. Because ensuring the homogeneity among all interfaces is difficult, it is natural that each magnetic layer has magnetic properties different from the others^11,12^. Static magnetic properties, such as the stable DW configuration, have been reported to significantly affect the dynamics of the DW motion^13^. For example, a stable DW configuration determines the direction of DW motion driven by the spin–orbit torque. Therefore, it is essential to understand the layer resolved DW configurations and their roles in DW dynamics in magnetic multilayered structures.

Most of the studies of magnetic DW motion in magnetic multilayered structures was done without consideration of the DW configuration. Only a few studies have focused on the DW configurations in magnetic multilayered structures^14,15^. Since the magnetic DW motion in magnetic multilayered structures have drawn more attention in magnetic application devices^1–4^, more comprehensive understanding on the magnetic DW motion is required within the context of the DW configuration.

In this study, we prepared magnetic multilayered structures composed of two magnetic layers and then investigated the DW dynamics in these structures using a magneto-optical Kerr effect microscope. Interestingly, the magnetic multilayered structures exhibit an exotic coupled behavior of the DW dynamics. A micromagnetic study was carried out to explain the exotic coupled behavior within the context of multiple Walker breakdowns of DWs in each magnetic layer.

## Results

### Double minimum of DW velocity about *H*_*x*_

**Figure 1:**
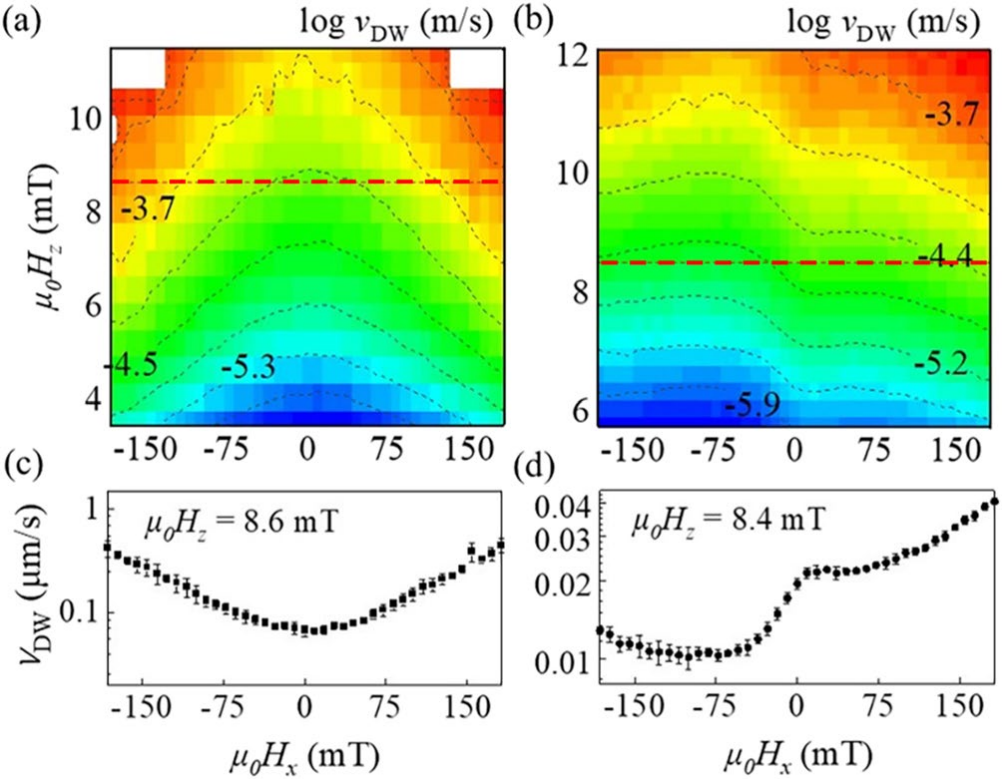
(**a**,**b**) 2-dimensional plots of $\log v_{{{\text{DW}}}}$ with respect to $H_{x}$ (abscissa) and $H_{z}$ (ordinate) for Samples I and II, respectively. (**c**,**d**) Line profiles of $v_{{{\text{DW}}}}$ with respect to $H_{x}$ along the horizontal lines in (**a**) and (**b**), respectively.

The DW displacements are measured along the direction (Fig. 5d, yellow arrow) parallel to *H*_*x*_, providing the DW speed, $v_{{{\text{DW}}}}$, in the direction of *H*_*x*_. Figure 1a,b plot the measured $v_{{{\text{DW}}}}$ onto the 2-dimensional (2D) coordinate plane with respect to *H*_*x*_ (abscissa) and *H*_*z*_ (ordinate) for Samples I and II, respectively. Sample I exhibits a typical symmetrical $v_{{{\text{DW}}}}$ variation with respect to *H*_*x*_. The line profile along the dashed red line in Fig. 1a is plotted in Fig. 1c, clearly confirming the symmetrical variation with a single minimum. Such symmetrical variation is known to be caused by the evolution of the stable DW configuration under the influence of *H*_*x*_^13^.

Interestingly, Sample II shows an exotic variation of $v_{{{\text{DW}}}}$ with two minima, as shown in the 2D plot of Fig. 1b and the line profile in Fig. 1d. Though it is well known that the asymmetry of $v_{{{\text{DW}}}}$ near $H_{x} \cong 0$ is ascribed to the Dzyaloshinskii–Moriya interaction^13,16^, the exotic variation with two minima goes well beyond the model based on a single DW configuration. To check whether the exotic variation with two minima is ascribed to the two magnetic layers as in Sample II, the coupled configuration between the DWs in the two magnetic layers was investigated by considering micromagnetism. The simulation results are discussed hereafter.

### Multiple walker breakdown

**Figure 2:**
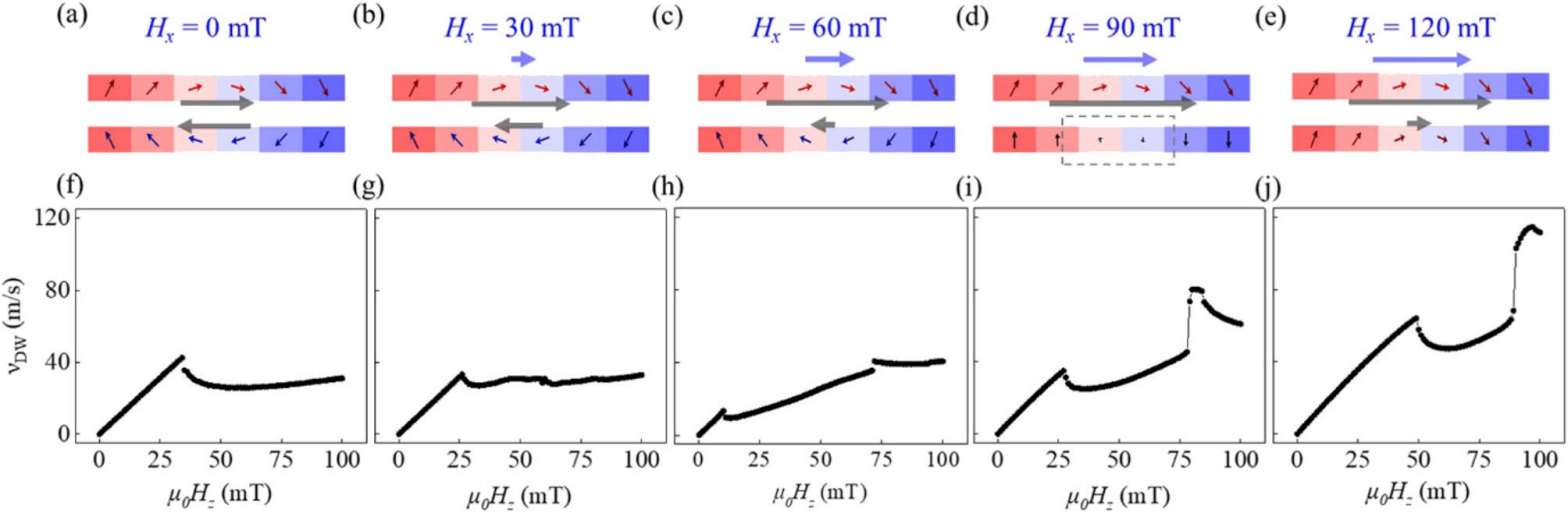
DW configurations under various $H_{x}$ values of (**a**) 0 mT, (**b**) 30 mT, (**c**) 60 mT, (**d**) 90 mT, and (**e**) 120 mT. The arrow inside each mesh shows the direction of the magnetization. The colors of arrows and meshes correspond to the *x* and z components of the magnetization, respectively. The gray arrows show *H*_tot_ at the upper and lower magnetic layer. The blue arrow shows the strength of $H_{x}$. (**f**–**j**) Plots of $v_{{{\text{DW}}}}$ with respect to $H_{z}$ for different $H_{x}$ values.

The stationary DW configurations at $H_{z} = 0$ were first examined under application of *H*_*x*_ over the range from 0 to 120 mT. Figure 2a–e shows the cross-sectional view of the stable DW configurations in the stationary state for different *H*_*x*_ values of (a) 0, (b) 30, (c) 60, (d) 90, and (e) 120 mT, respectively. The arrow inside each mesh shows the direction of the magnetization. The color of each arrow corresponds to the $x$ component of the magnetization (red for + *x* direction and blue for − *x* direction), whereas the color inside each mesh corresponds to the z component of the magnetization (red for + *z* direction and blue for − *z* direction).

When $H_{x} = 0$, as shown in Fig. 2a, the dipolar magnetic field from the lower magnetic layer generates a local magnetic field, $H_{{{\text{dip}}}}$. For the present geometry, such $H_{{{\text{dip}}}}$ has a net in-plane component along the + *x* direction (upper gray arrow) at the position of the DW in the upper magnetic layer. Thus, the upper DW becomes stabilized as the Néel-type configuration with internal magnetization along the + *x* direction. Inversely, the dipolar magnetic field from the upper magnetic layer generates a net $H_{{{\text{dip}}}}$ along the − *x* direction (lower gray arrow) at the lower DW, thus resulting in the opposite Néel-type configuration along the − *x* direction. Consequently, an antiparallel alignment of the internal magnetization appears between the upper and lower DWs, as shown in Fig. 2a.

Under the application of *H*_*x*_ (blue arrows), the total in-plane magnetic field, $H_{{{\text{tot}}}}$ ($= H_{x} + H_{{{\text{dip}}}}$) becomes unbalanced at positions between the upper and lower DWs. For the present geometry, an increase in *H*_*x*_ causes the decrease of *H*_tot_ at the lower DW, as shown by the lower gray arrows in Fig. 2b,c. When *H*_tot_ vanishes, the DW becomes a Bloch-type configuration, as shown in Fig. 2d. A further increase in *H*_*x*_ results in the parallel alignment of the internal magnetization between the upper and lower DWs, as shown in Fig. 2e.

To examine the effect of these different DW configurations, the DW speed, $v_{{{\text{DW}}}}$ is calculated under the application of *H*_*z*_. Figure 2f–j plot $v_{{{\text{DW}}}}$ with respect to *H*_*z*_ for different *H*_*x*_ values. When $H_{x} = 0$, as shown in Fig. 2f, $v_{{{\text{DW}}}}$ exhibits an abrupt breakdown at a certain strength of *H*_*z*_. This breakdown is well known as the Walker breakdown, which is caused by the precession of the internal magnetization inside the DWs.

### Phases of DW during DW motion

**Figure 3:**
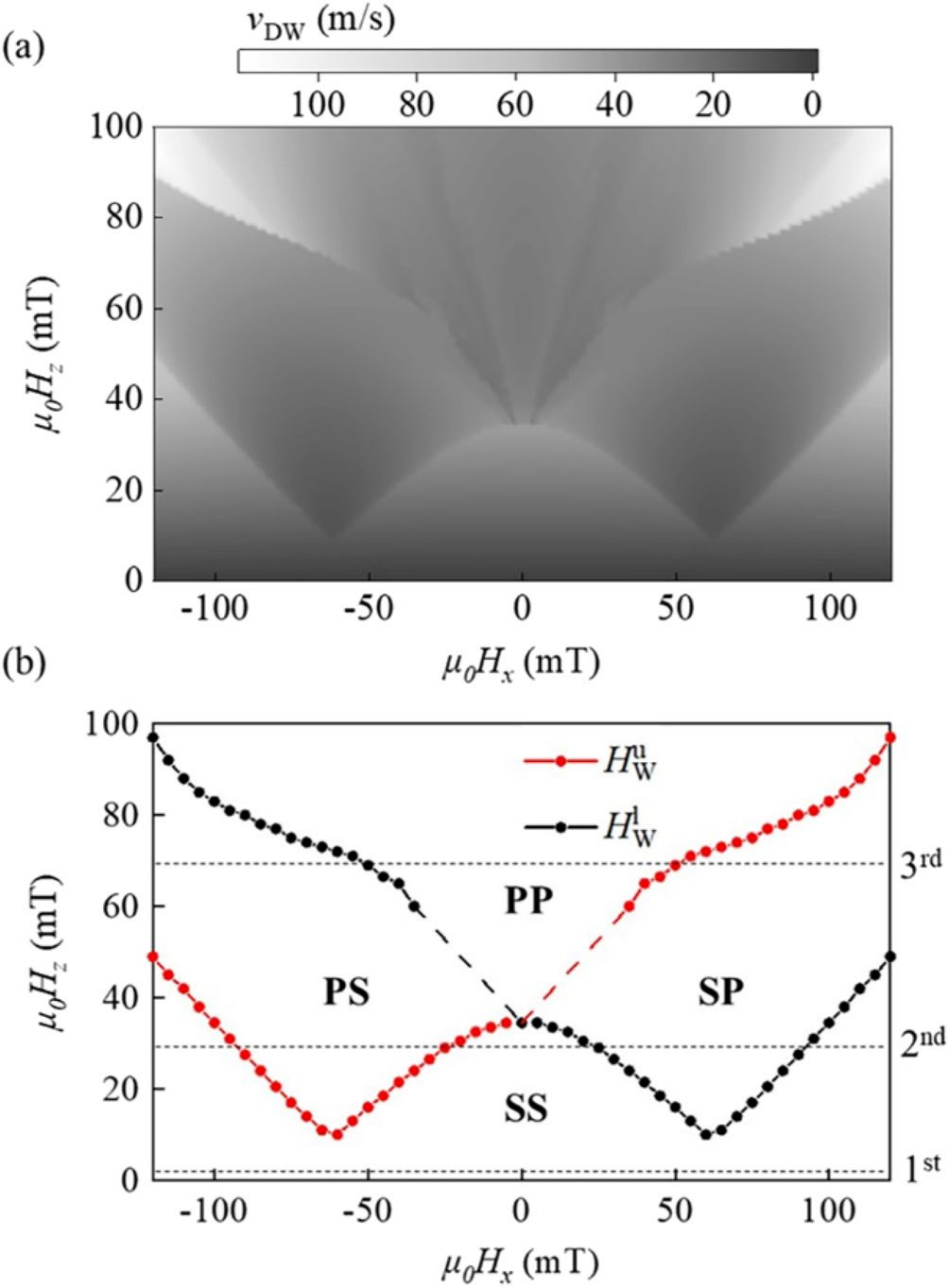
(**a**) 2-dimensional plots of the calculated $v_{{{\text{DW}}}}$ with respect to $H_{x}$ (abscissa) and $H_{z}$ (ordinate). (**b**) Phase diagram of the PP, PS, SP, and SS phases. The red and black lines indicate the Walker breakdown fields, $H_{{\text{W}}}^{{\text{u}}}$ and $H_{{\text{W}}}^{l}$, of the upper and lower layers, respectively. The horizontal lines are the paths of the line profiles shown in Fig. 4.

Figure 3a shows a 2D plot of $v_{{{\text{DW}}}}$ with respect to *H*_*x*_ (abscissa) and *H*_*z*_ (ordinate). It is clear from the plot of several different phases with phase boundaries. The phase boundaries are attributed to the Walker breakdown, below which a steady-state motion with a fixed magnetization angle appears, above which a precessional motion occurs. For better visualization, Fig. 3b illustrates the four phases: phase SS of steady-state motions (both DWs), phase PS of precessional (upper DW) and steady-state (lower DW) motions, phase SP of steady-state (upper DW) and precessional (lower DW) motions, and phase PP of precessional motions (both DWs). The phase boundaries indicate the Walker breakdown fields, $H_{{\text{W}}}^{{\text{u}}}$ (red symbols) and $H_{{\text{W}}}^{l}$ (black symbols), for the upper and lower DWs, respectively.

Inside the phase diagram, the line profiles (horizontal dashed lines) under a fixed *H*_*z*_ provide the $v_{{{\text{DW}}}}$ variation with respect to *H*_*x*_, similar to the experimental measurement procedure, shown in Fig. 1d.

### DW velocity by walker breakdown

**Figure 4:**
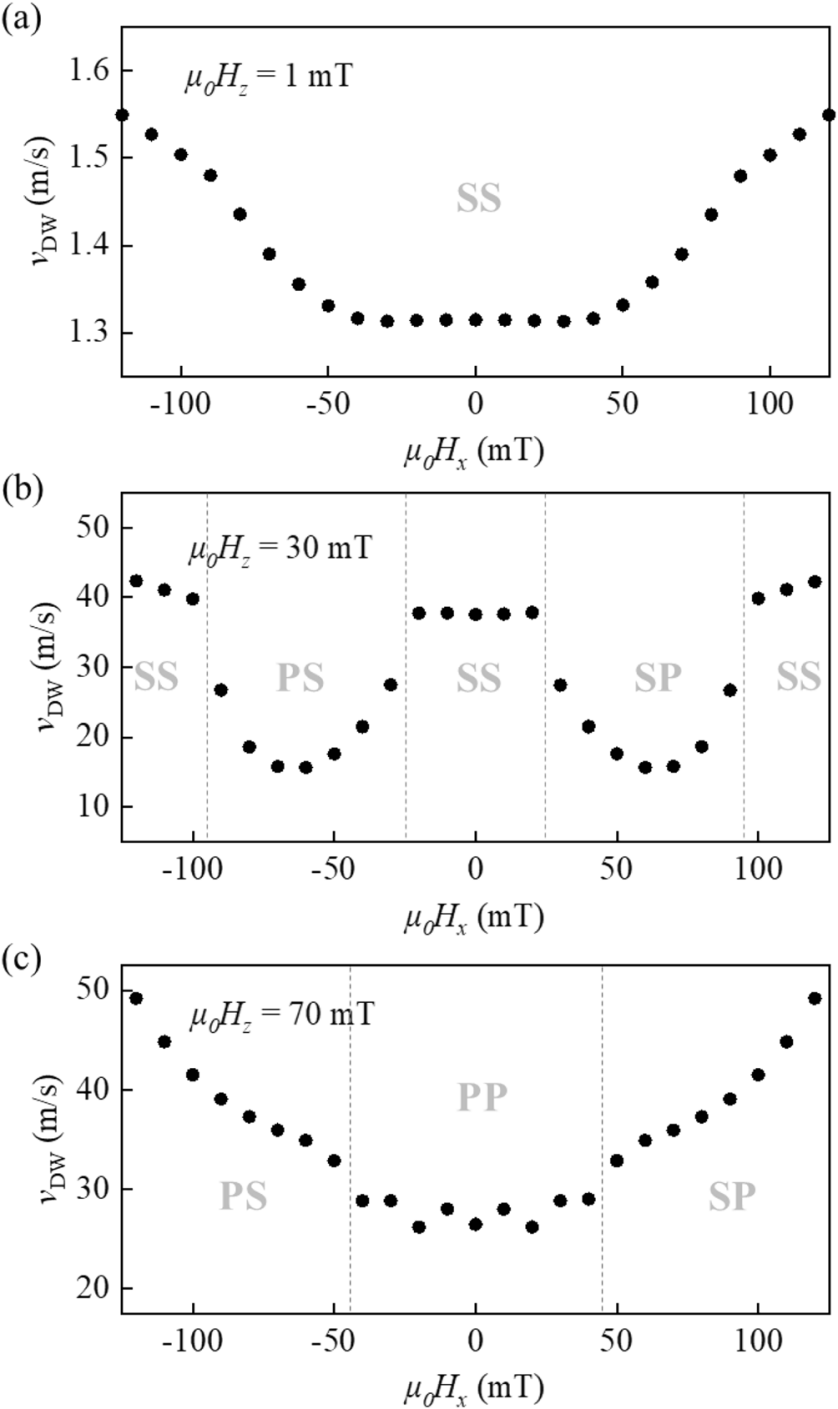
Line profiles of $v_{{{\text{DW}}}}$ with respect to $H_{x}$ under fixed $H_{z}$ of (**a**) 1, (**b**) 30, and (**c**) 70 mT. The paths of the line profiles are shown by the horizontal lines in Fig. 3b.

Figure 4a–c plot the $v_{{{\text{DW}}}}$ variation with respect to *H*_*x*_ under several fixed *H*_*z*_ values. Here, the presents $v_{{{\text{DW}}}}$ variations can be considered as the global $v_{{{\text{DW}}}}$ of the bilayer (as seen in Fig. 2) and then, be explained by the combined effect of the DWs of the lower and upper layers (as seen in Fig. 3). For a weak *H*_*z*_, the line profile along the first horizontal dashed line in Fig. 3b stays inside phase SS for both DWs. The $v_{{{\text{DW}}}}$ variation almost exhibits a plateau in the middle with respect to *H*_*x*_, as shown in Fig. 4a. However, a closer observation showed two separated minima with small amplitudes. These separated minima become obvious for an intermediate *H*_*z*_, where the line profile along the second horizontal, dashed line in Fig. 3b crosses phases PS, SS, and SP. Because $v_{{{\text{DW}}}}$ in phases PS and SP is largely reduced owing to the Walker breakdown, the $v_{{{\text{DW}}}}$ variation naturally exhibits two large minima inside these phases, as shown in Fig. 4b. Finally, for a strong *H*_*z*_, the line profiles along the third horizontal dashed line in Fig. 3b cross phases PS, PP, and SP. Because the $v_{{{\text{DW}}}}$ of phase PP is slower than those of the other phases owing to the Walker breakdowns for both DWs, its $v_{{{\text{DW}}}}$ variation exhibits a single minimum at the middle, that is, inside phase PP, as shown in Fig. 4c. Notably, the experimental value of *H*_*z*_ in Fig. 1b ranges between those values shown in Fig. 4a,b. Therefore, the double Walker breakdowns for each DW of the two magnetic layers can explain the experimental observation of the exotic $v_{{{\text{DW}}}}$ variation.

## Discussion

The magnetic field for the breakdown is denoted as the Walker breakdown field, $H_{{\text{W}}}$. According to Ref.^17^, $H_{{\text{W}}}$ follows the relation, $H_{{\text{W}}} = \alpha \sin \psi_{{\text{W}}} \left( {\left| {H_{{{\text{tot}}}} } \right| - H_{{{\text{DW}}}} \cos \psi_{{\text{W}}} } \right)$, where $H_{{{\text{DW}}}}$ is the DW anisotropy field. Here, azimuthal angle $\psi_{{\text{W}}}$ of the magnetization inside the DW is defined as $\cos \psi_{{\text{W}}} = \left( {\left| {H_{{{\text{tot}}}} } \right| - \sqrt {H_{{{\text{tot}}}}^{2} + 8H_{{{\text{DW}}}}^{2} } } \right)/4H_{{{\text{DW}}}}$.

In addition, when $H_{x} = 0$, both the upper and lower DWs have the same $\left| {H_{{{\text{tot}}}} } \right|$ value, resulting in identical strengths of $H_{{\text{W}}}$. Therefore, both DWs exhibit a single Walker breakdown simultaneously, as shown in Fig. 2f. However, under the application of $H_{x}$, the upper and lower DWs have different values of $\left| {H_{{{\text{tot}}}} } \right|$, resulting in different strengths of $H_{{\text{W}}}$. This could possibly result in two separate Walker breakdowns. As $H_{x}$ increases, such a separation becomes more obvious and dominant, as shown in Fig. 2g–j.

In summary, we investigated the phases of the DW motion among two magnetic layers. The micromagnetic simulation results reveal that each magnetic layer has a different internal dipolar magnetic field, and thus the DW in each magnetic layer experiences different magnetic fields. Owing to the different Walker breakdown fields for each DW, the $v_{{{\text{DW}}}}$ variation exhibits two minima with respect to $H_{x}$. The presented simulation results satisfactorily explain the experimental observation of the two minima in the $v_{{{\text{DW}}}}$ variation.

## Methods

### Sample preparations and DW velocity measurements

**Figure 5:**
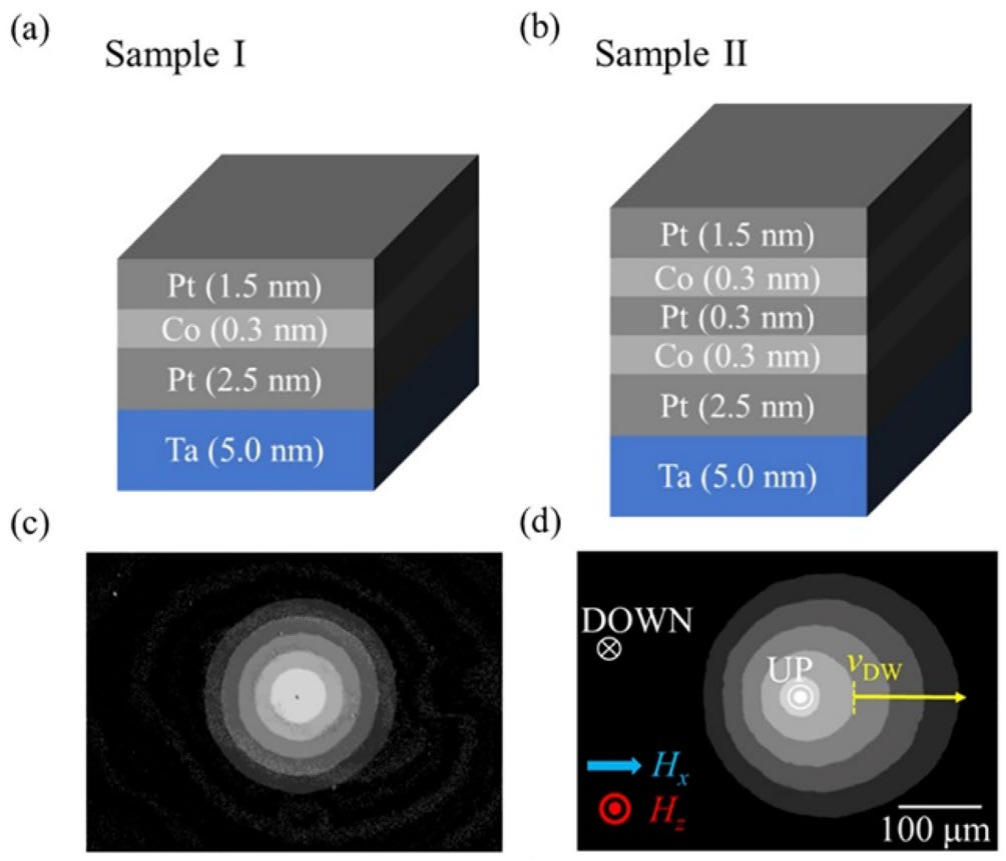
(**a**,**b**) Layer structures of Samples I and II, respectively. (**c**,**d**) Magnetic domain expansion images overlaid with the snapshots taken at a specified time interval for Samples I and II, respectively. The blue arrow and red symbol represent directions of $H_{x}$ and $H_{z}$, respectively. The white symbols indicate the directions of magnetization. The brightest circle in the middle represents the initial magnetic domain. The yellow arrow indicates the direction of the $v_{{{\text{DW}}}}$ measurement.

For this study, we prepared two types of magnetic films with different numbers of magnetic layers. Here, we denote Samples I and II for films composed of a single magnetic layer and double magnetic layers, respectively. The detailed layered structures comprise 5-nm Ta/2.5-nm Pt/0.3-nm Co/1.5-nm Pt for Sample I and 5-nm Ta/2.5-nm Pt/0.3-nm Co/0.3-nm Pt/0.3-nm Co/1.5-nm Pt for Sample II, as illustrated in Fig. 5a,b, respectively. The films were deposited on Si wafers with a 100-nm SiO_2_ layer using dc magnetron sputtering.

Both films exhibited strong perpendicular magnetic anisotropy and clear magnetic domain patterns. Figure 5c,d shows the magnetic domain images of Samples I and II, respectively. Each image is overlaid with several snapshots taken by magneto-optical Kerr effect (MOKE) microscopy at a specified time interval under the application of the out-of-plane magnetic field, $H_{z}$ (red symbol), together with the in-plane magnetic field, $H_{x}$ (blue arrow)^13^. The brightest circle in the middle represents the initial circular magnetic domain, which exhibits gradual expansion over time, as shown by the gradual change in image contrast.

### Micromagnetic simulation

We employed the object-oriented micromagnetic framework (oommf) code in three-dimensional geometry. The simulation geometry was set as a lateral wire structure onto the film of two ferromagnetic (FM) layers separated by a nonmagnetic (NM) spacer. The lateral wire structure has a length of 2000 nm and a width of 250 nm. All FM and NM layers have an identical thickness of 0.4 nm. The mesh size was chosen to be 1 nm along the length, 250 nm along the width, and 0.4 nm along the thickness. For a fast simulation speed with minimal number of meshes, the mesh size along the width is set to be identical to the wire width. The mesh size along the length was set to be sufficiently small to describe the internal DW structures. The magnetic parameters are chosen as the typical values of Co/Pt multilayers, as given by the exchange stiffness of 5 × 10^–12^ J/m, saturation magnetization of 1.4 × 10^6^ A/m, and surface magnetic anisotropy of 1.93 × 10^6^ J/m^3^^18^. For fast relaxation to the ground state, the damping constant was set at $\alpha$ = 0.5.

## Data Availability

The datasets generated during and/or analysed during the current study are available from the corresponding author on reasonable request.
